# Supplementary material for: ERK1/2 Inhibition via the Oral Administration of Tizaterkib Alleviates Noise-Induced Hearing Loss While Tempering down the Immune Response
Source: Int J Mol Sci. 2024 Jun 7;25(12):6305. doi: 10.3390/ijms25126305 (PMC11204379; doi:10.3390/ijms25126305)
Supplement: Supplementary file 1 [file ijms-25-06305-s001.zip › ijms-3039944-supplementary.pdf]

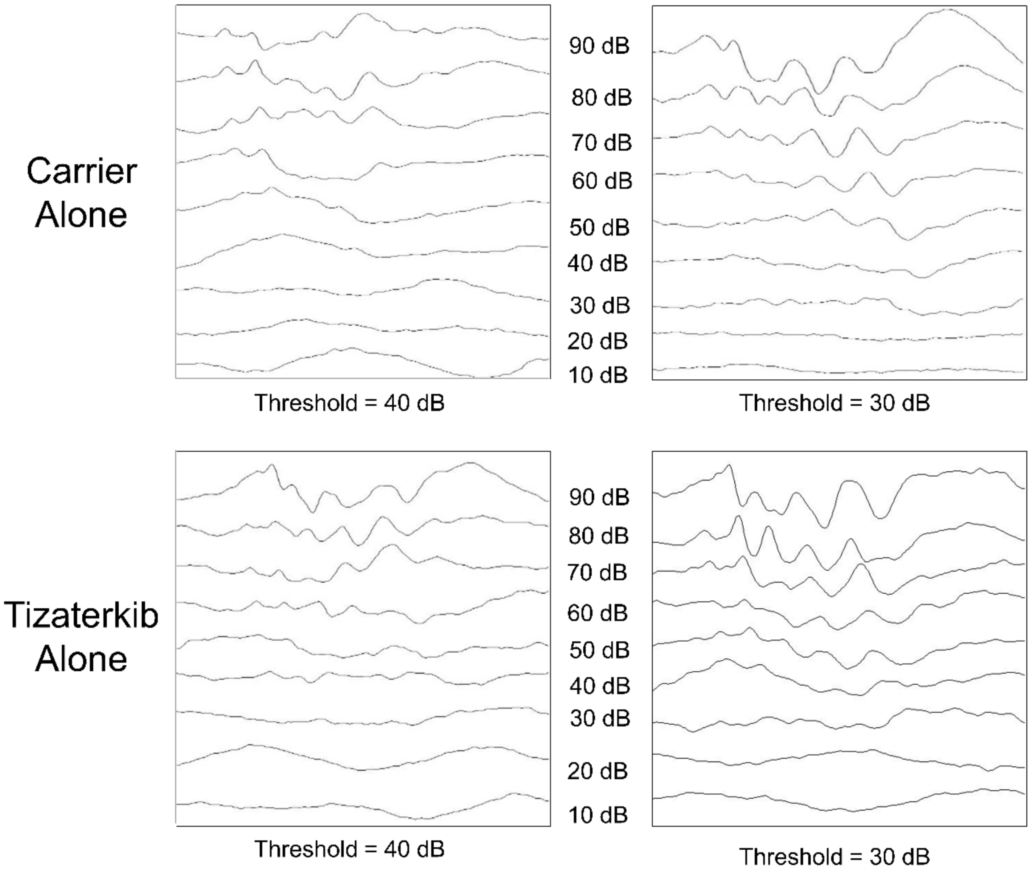

<u>Supplemental Figure S1:</u> Representative ABR traces. Representative post treatment ABR traces of carrier and tizaterkib alone treated mice are shown from data in Figure 2. The threshold was recorded as the last trace with at least 3 of the 5 ABR waveforms present.

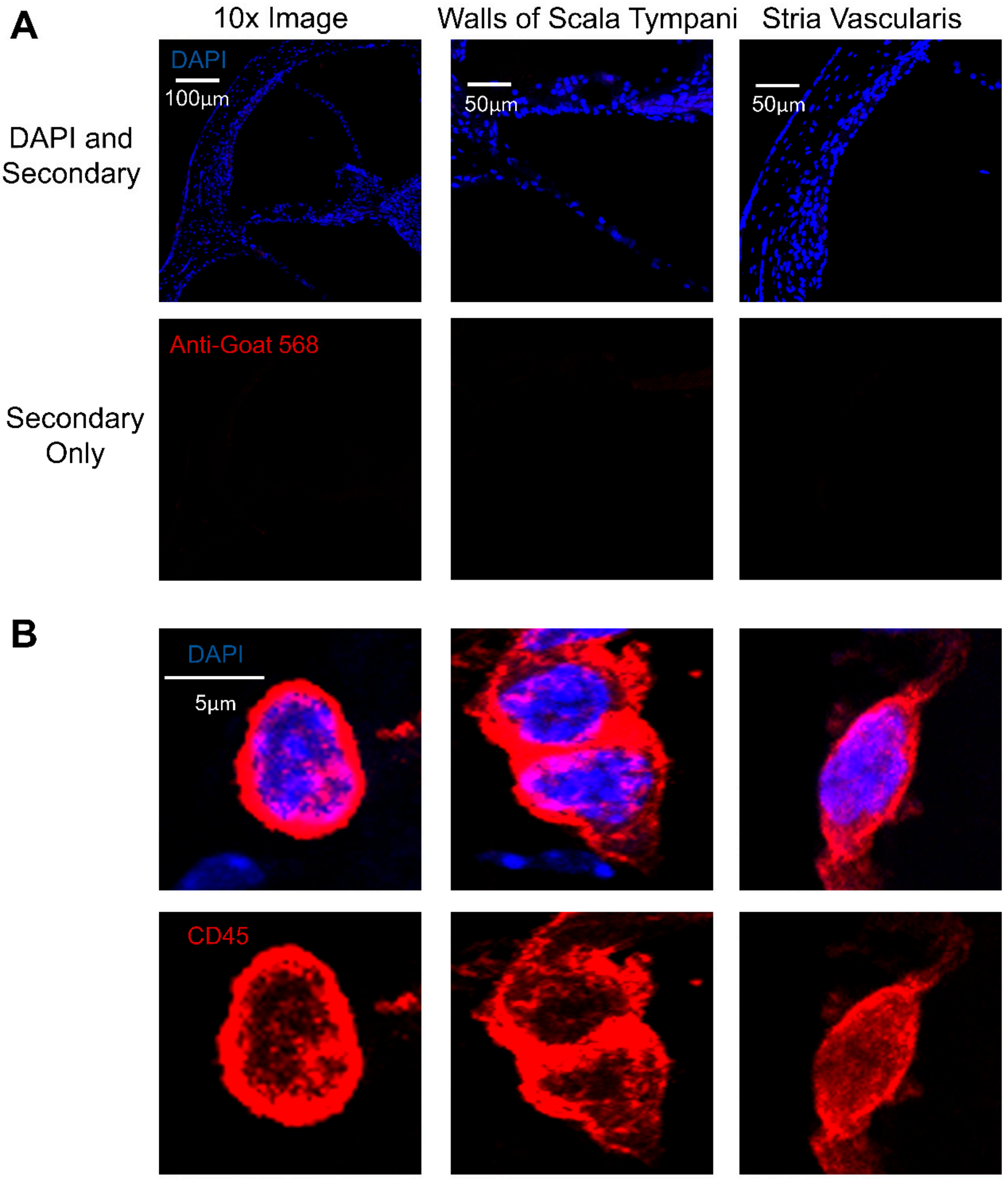

<u>Supplemental Figure S2:</u> No positive immunostaining occurred with secondary alone and zoomed in images of CD45 positive cells. (A) Representative images of cochlear cryosections stained with DAPI (blue) and 568 donkey anti-goat secondary antibody (red) (secondary by itself with no CD45 primary antibody). Top image is DAPI and secondary together

and bottom image is secondary by itself. **(B)** Zoomed in images of CD45 positive cells. Top image is DAPI (blue) and CD45 (red) together and bottom image is CD45 by itself.

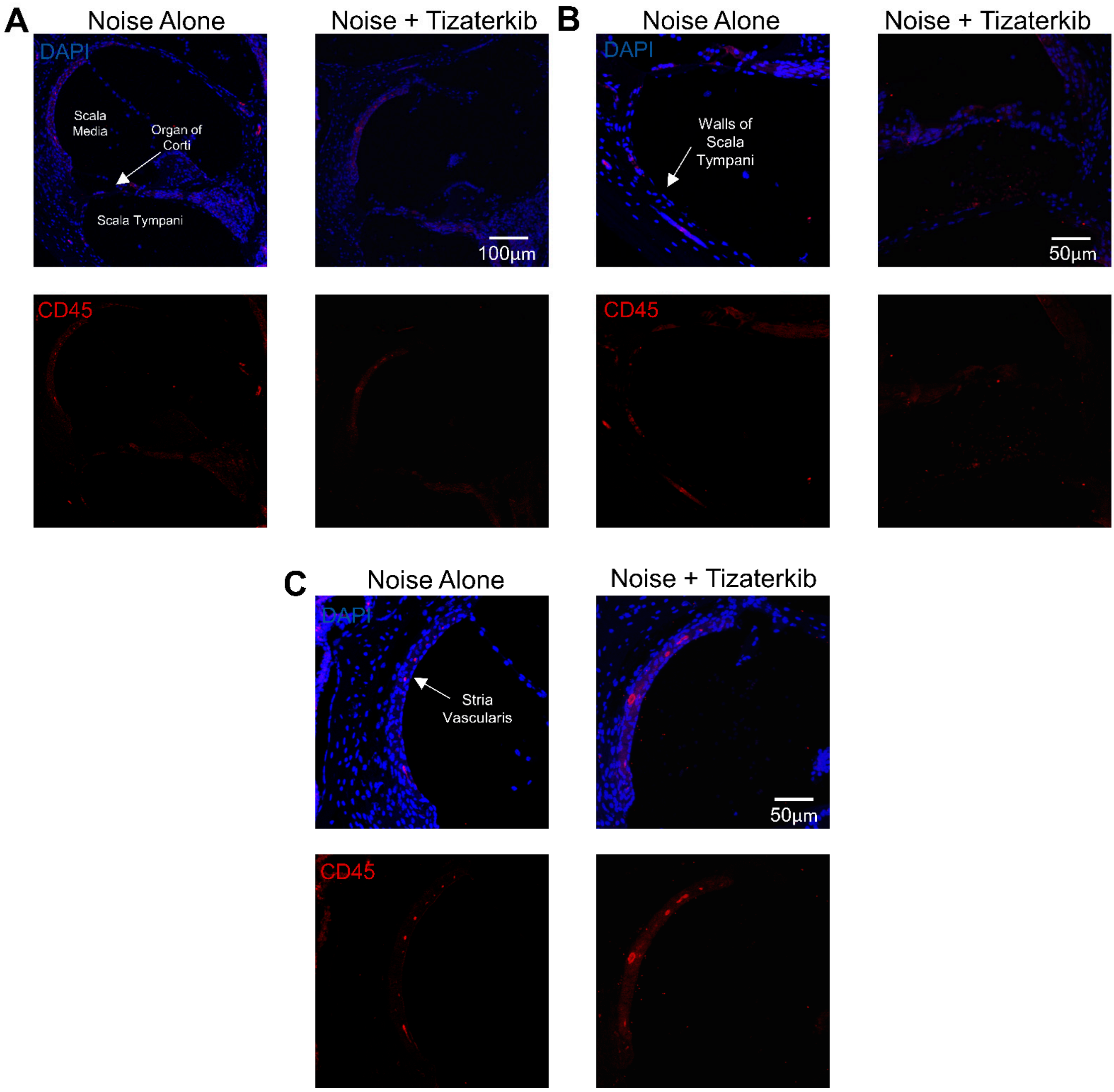

<u>Supplemental Figure S3:</u> The number of CD45 positive cells are back to baseline levels 8 days following noise exposure. (A) Representative images of cochlear cryosections of noise and noise + tizaterkib treated mice 8 days following noise exposure. Sections are stained with DAPI (blue) and CD45 (red). Image on top has DAPI and CD45 staining and image below has CD45 alone. **(B)** Zoomed in representative images of the scala tympani region 8 days after noise exposure. **(C)** Zoomed in representative images of the stria vascularis 8 days after noise exposure.
